# PYCR1: A Potential Prognostic Biomarker in Pancreatic Ductal Adenocarcinoma

**DOI:** 10.7150/jca.61498

**Published:** 2022-02-28

**Authors:** Huanyu Wang, Weilin Mao, Wenhui Lou, Dayong Jin, Wenchuan Wu, Dansong Wang, Tiantao Kuang, Yefei Rong, Xuefeng Xu, Lei Zhang

**Affiliations:** 1 Department of General Surgery, Xuhui District Central Hospital, Shanghai, China.; 2 Department of General Surgery, Zhongshan Hospital, Fudan University, Shanghai, China.; 3 Cancer Center, Zhongshan Hospital, Fudan University, Shanghai, China.

**Keywords:** PYCR1, Pancreatic ductal adenocarcinoma, Prognosis

## Abstract

**Background/Objectives:** Pancreatic ductal adenocarcinoma (PDAC) is a highly aggressive malignant tumor with an extremely poor prognosis in digestive tumors. Pyrroline-5-carboxylate reductase 1 (PYCR1) plays an important role in tumor development. Therefore, we aimed to explore the effect of PYCR1 on the growth of PDAC cells.

**Methods:** Tumor tissues and adjacent normal pancreatic tissues were collected from 89 patients with PDAC. And immunohistochemistry (IHC) was used to analyze the expression level of PYCR1 in both. RNA interference was used to inhibit the expression of PYCR1 in PANC- 1 and AsPC-1 cells. After infection, the expression of PYCR1 protein was detected by Western blot. The proliferation and growth of PDAC cells were detected by Celigo analysis, MTT, and clone formation assay. Cell apoptosis was analyzed by flow cytometry. Furthermore, the effect of PYCR1 interference on tumor growth was evaluated *in vivo* through injecting tumor cells subcutaneously into nude mice.

**Results:** The expression of PYCR1 in pancreatic cancer tissues was significantly higher than in paired adjacent normal pancreatic tissues (P <0.01). *In vitro*, the downregulation of PYCR1 expression significantly inhibited the cell proliferation and colony formation, and increased apoptosis in PANC-1 cells and AsPC-1 cells compared with the shCtrl group (P <0.01). And *in vivo*, PYCR1 interference also significantly inhibited tumor growth both in the tumor volume and weight.

**Conclusion:** PYCR1 interference was able to inhibit cell proliferation and promote cell apoptosis of pancreatic cancer. The PYCR1 may serve as a potential therapeutic and prognostic biomarker for the treatment of pancreatic cancer.

## Introduction

Pancreatic cancer is a highly malignant tumor with poor diagnosis in the digestive system. Pancreatic cancer is occult without specific symptoms, which means that many patients exhibit locally advanced disease or metastasis at the time of diagnosis 1,2. Pancreatic ductal adenocarcinoma (PDAC), originating from the ductal epithelium of the pancreas, accounts for more than 90% of all pancreatic cancer cases. Currently, surgical resection is the only chance of cure for early-stage PDAC 3. Only 15-20% of pancreatic cancer is resectable 4. Meanwhile, a high mortality rate is due to late diagnosis. However, the prognosis of patients with resectable tumors remains poor, with a 5-year survival rate of around 20% 5-7. Radiotherapy and chemotherapy are still the main treatments for unresectable pancreatic cancer, but the effects of radiotherapy and chemotherapy are not satisfactory 3,8,9. Therefore, there is an urgent need to explore effective diagnostic, therapeutic, and prognostic targets for patients with pancreatic cancer.

Pyrroline-5-carboxylate reductase (PYCR) is mainly located in the mitochondria and plays an important role in the biosynthesis of proline 10,11. PYCR catalyzes an NADPH dependent reaction of pyrroline-5-carboxylate (P5C) into proline. Furthermore, there are three PYCR isoenzymes (PYCR1, PYCR2, PYCRL) in human tissues. And defects in the PYCR1 gene can lead to the abnormality of mitochondrial membrane potential, which results in mitochondrial network disruption and the apoptosis rate increasing during oxidation 12-15. PYCR1 has been reported to play a key role in the development of bone, connective tissues, and fat tissues, mutation of which can lead to cutis laxa 16-18.

As non-essential amino acids in the human body, proline has a unique secondary amine structure, and enzymes involved in the metabolism of proline play a key role in the control of tumor initiation and progression. According to previous reports, PYCR1, one of the most important enzymes in the process of proline metabolism, is closely associated with tumorigenesis and tumor progression, including prostate cancer, breast cancer, colon cancer, lung cancer, and liver cancer 19-23. Hence, PYCR1 may be a potential target for early diagnosis and therapeutic intervention. However, the effect and mechanism of PYCR1 in pancreatic cancer remain unknown.

In this study, we aim to analyze the correlation between PYCR1 and PDAC. The expression level of PYCR1 was evaluated in pancreatic cancer. And we explored the biology behavior of PYCR1 both *in vitro* and *in vivo*.

## Materials and methods

### Patients and samples

This study involved 89 tumor tissues and paired adjacent normal pancreatic tissues from patients who underwent curative surgical resection in Zhongshan Hospital, Fudan University from September 2012 to May 2016. Tissues were collected during surgery and frozen in liquid nitrogen. All specimens were pathologically confirmed to be ductal adenocarcinoma of the pancreas. And all the clinical pathological data and detailed follow-up data of the 89 patients were collected. Patients were followed up until May 1, 2020. Furthermore, the tissues were used for the analysis of immunohistochemical (IHC). This study was approved by the Ethics Committee of Zhongshan Hospital, Fudan University. All patients signed informed consent before the operation.

### Immunohistochemistry (IHC)

#### Staining method

All pancreatic tissues were fixed in 10% formalin and embedded in paraffin. The sections were dewaxed with xylene and dehydrated with ethanol. After deparaffinization, the sections were treated with 3% hydrogen peroxide for 10 minutes. For antigen retrieval, the sections were heated in sodium citrate buffer (pH 6.0) for 20 minutes. After that, sections were cooled to room temperature naturally and dipped in distilled water for 10 min. Sections were blocked with 10% fetal bovine serum (FBS) for 30minutes and incubated overnight with primary rabbit polyclonal anti-PYCR1 antibody (1:100, Abcam, ab103314). Then, the second antibody and Vulcan Fast Red Chromogen kit2 was added. Sections were stained with diaminobenzidine (DAB) and were counterstained with hematoxylin. The results were observed under an optical microscope after dehydration and sealing.

#### Interpretation standards

The brown staining in the cytoplasm indicated positive staining for PYCR1. The tissues were evaluated by two different pathologists. The expression of PYCR 1 staining was scored according to the following criteria independently.

(1) The percentage of positive staining was scored as follows: 0, 0-5% positively stained cells; 1, 6-20% positively stained cells; 2, 21-50% positively stained cells; 3, 51-100% positively stained cells.

(2) The intensity of staining was scored as follows: 0, negative; 1, weakly positive; 2, moderately positive; 3, strongly positivity.

(3) The histochemistry score (H-score, 0-9) was calculated by multiplying the percentage of positive staining score with the staining intensity score. Then, the tissues were divided into two groups: low expression (score≤3) and high expression (score >3).

### Cell culture

The pancreatic cancer cell lines used in this study include SW1990, PANC-1, AsPC-1, BxPc-3, which were purchased from the Chinese Academy of Sciences Cell Bank (Shanghai, China). These cells were cultured in DMEM media containing 10% FBS at 37 °C with 5% CO_2_.

### RNA interference of PYCR1 by lentivirus

Small-hairpin RNA (shRNA) interfering sequences targeting PYCR1 (shPYCR1: 5'-CAGTTTCTGCTCTCAGGAA-3') and negative shRNA sequences (shCtrl: 5'-TTCTCCGAACGTGTCACGT-3') were designed and synthesized. After that, the single-stranded DNA oligo containing the interference sequence was synthesized and converted to double-stranded DNA by annealing. And GV115 vector (Shanghai Genechem Co., Ltd, Shanghai, China) was linearized with Age I and EcoR I restriction enzymes. After digestion, the double-stranded DNA oligo was ligated to the GV115 vector using T4 DNA Ligase (Fermentas) according to the manufacturer's instructions. Then, the products were transformed into the E. coli TOP10 strain (TIANGEN Biotech Co., Ltd, Shanghai, China). The positive clones were identified and selected by sequencing. According to the manufacturer's instructions of EndoFree Maxi Plasmid Kit (TIANGEN Biotech Co., Ltd, Shanghai, China), the plasmid was extracted from the previous positive clones for further lentiviral packaging.

The AsPC-1 and PANC-1 cells were seeded in a 6-well plate at a density of 2×10^5 per well and infected with recombinant PYCR1 (shPYCR1) or negative control (shCtrl) lentivirus separately (MOI of 20). At 72 hours post infection, the fluorescence signals of green fluorescent protein (GFP) were observed with a fluorescence microscope (IX71, Olympus, Tokyo, Japan). Only cells, in a well-growing state and positive for GFP fluorescence over 70%, were collected for further detection. And fluorescence-activated cell sorting (FACS, BD Bioscience Aria II) was used to sort for GFP-positive cells.

### Real-time quantitative polymerase chain reaction (RT-qPCR)

For the examination of PYCR1 expression, total RNA was extracted from the cell lines using TRIzol reagent (Pufei Biotech Co., Ltd, Shanghai, China). And total RNA was reverse transcribed into cDNA using M-MLV reverse transcriptase kit (Promega). The RT-qPCR was performed using SYBR® Premix Ex Taq™ (Takara Biotechnology Co., Ltd) in a volume of 12μL on LightCycler 480 II Real-Time PCR System (Roche). PCR cycling conditions were as follows: 95 °C for 30s, 40 cycles at 95 °C for 5 s, 60 °C for 30s. GAPDH was used as an internal control to calculate the relative expression levels of genes. The relative mRNA expression levels were calculated by the 2-ΔΔCT method. All samples were assayed in triplicate.

The primers used in this study were listed as follows.

PYCR1-forward (F): 5'-GGCTGCCCACAAGATAATGGC-3';

PYCR1-reverse (R): 5'-CAATGGAGCTGATGGTGACGC-3';

shPYCR1-F: 5'-TTGGCTGCCCACAAGATAAT-3';

shPYCR1-R: 5'-ATCACTGTGCTGCACCGTCT-3';

GAPDH-F: 5'-TGACTTCAACAGCGACACCCA-3';

GAPDH-R: 5'-CACCCTGTTGCTGTAGCCAAA-3'.

### Cell proliferation assay

The AsPC-1 and PANC-1 cells infected by shPYCR1 or shCtrl lentivirus were seeded in a 96-well plate in triplicate at a density of 1.5×10^3 per well. From the second day after seeding, the cells with GFP fluorescence were counted by the Celigo Imaging Cytometer (Nexcelom Bioscience, Lawrence, MA, USA) for 5 days. And the cell proliferation assay is performed at specific times.

Further, MTT assays were performed to examine the effect of PYCR1 on cell viability and proliferation. MTT assay began from the second day after seeding. MTT solution (5 mg/ml, 20 μl/well) was added to each well and incubated for 4 hours at 37 °C. After that, the MTT solution was removed. 100 μl dimethyl sulfoxide (DMSO) was added to dissolve the formed formazan crystals. The absorbance at 490 nm was measured by a microplate reader (Infinite M2009PR, Tecan). Three independent sets of experiments were performed at least.

### Apoptosis detection assay

Cell apoptosis assay was performed by the Annexin V-APC Apoptosis Detection Kit (eBioscience, San Diego, CA, USA) following the manufacturer's instructions. The PANC-1 and AsPC-1 cells infected by shPYCR1 or shCtrl lentivirus were seeded in 6-well culture plates and cultured for 5 days. The adherent cells were digested with trypsin, washed with PBS, and centrifuged. The cell suspension was mixed with Annexin V-APC and incubated for 10 minutes protected from light. The data were collected and analyzed using a flow cytometer with CellQuest software (Becton-Dickinson, San Jose, CA, USA). Three independent sets of experiments were performed at least.

### Colony formation assay

The colony formation assay was performed to assess the effect of PYCR1 on cell survival. The cells infected by the lentivirus were cultured for 5 days and then digested into cell suspension with trypsin. After digestion, the cells were seeded in 6-well culture plates at a density of 2,000 per well. After cultured for 14 days, the cells were fixed with 4% paraformaldehyde and stained with Giemsa solution (Dingguo Biotech Co., Ltd, Shanghai, China). The colonies were counted on a fluorescence microscope. Three independent sets of experiments were performed at least.

### Western Blot analysis

The infected cells were collected, washed twice with PBS, and lysed in lysis buffer. After lysed on ice for 15 min, the cells were broken by sonication. Then, the samples were centrifuged at 10000 g for 15 min at 4 °C to collect the supernatant. The protein concentration was measured using a bicinchoninic acid (BCA) protein assay kit (Beyotime Biotech Co., Ltd, Shanghai, China). The proteins were separated by 10% SDS-polyacrylamide gel and electro-transferred onto a polyvinylidene fluoride (PVDF) membrane (Millipore, Bedford, MA, USA). The membranes were blocked with 5% non-fat milk in TBST for 1 hour at room temperature and incubated with primary antibody overnight at 4 °C. And then the samples were incubated with the respective second antibody (1:2,000) for 1.5 hours. The protein signal was visualized using the enhanced chemiluminescence kit (ECL-plus; Thermo). GAPDH was used as an internal control.

The antibodies used in this study were as follows: Anti-PYCR1 (No. ab103314; 1:300; Abcam, CA, USA), Anti-GAPDH (No. sc-32233; 1;2,000; Santa Cruz, TX, USA), Anti-Mouse IgG (No. #7076; 1:2,000; Cell Signaling Technology, CA, USA), Anti-Rabbit IgG (No. #7074; 1:2,000; Cell Signaling Technology, CA, USA).

### *In vivo* tumor growth experiment

Twenty Balb/c nude mice (female, 4 weeks, 15-20g, NO: SCXK2018-0003) were purchased from Shanghai Lingchang Biotechnology to assess the effect of PYCR1 *in vivo*. All mice were randomly divided into two groups, normal control group (NC group, n=10) and knockdown group (KD group, n=10). AsPC-1 cells (5×10^6 cells) infected with shPYCR1 or shCtrl lentivirus were subcutaneously inoculated into the right flank of the nude mice. Data collection started 1 week after the injection. The mice were weighed and tumor volumes were measured twice per week. The tumor volume was calculated by the formula: Tumor volume = π/6 × Length × Width × Width. After 28 days, all mice were euthanized, and the tumors were removed and weighed. All experimental protocols involving animal experiments were approved by the Ethics Committee of Zhongshan Hospital, Fudan University.

### Statistical analysis

In this study, all statistical analyses were performed by the SPSS software version 26.0 (IBM, USA) and the GraphPad Prism software version 8.0 (GraphPad, USA). The comparison between the means of two groups was analyzed by the F-test to test the homogeneity of variance, followed by paired-samples t-test or Student's t-test when appropriate. A Pearson's chi-square test, Fisher's exact test were used to compare the proportions. Survival analyses were performed by the Kaplan-Meier survival analysis and the overall survival (OS) was calculated from the time of diagnosis until tumor-related death. The log-rank test was used to compare the survival curves. Univariable and multivariable Cox regression analysis were used to identify independent factors.

Risk factors are expressed as the hazard ratio [HR, 95% confidence interval (CI)]. p < 0.05 was considered statistically significant (*, P < 0.05; **, P < 0.01). All experiments were conducted three times and the error bars indicate the standard deviation from the mean of triplicate measurements.

## Results

### Elevated PYCR1 expression in PDAC tissues and cell lines

**Figure 1:**
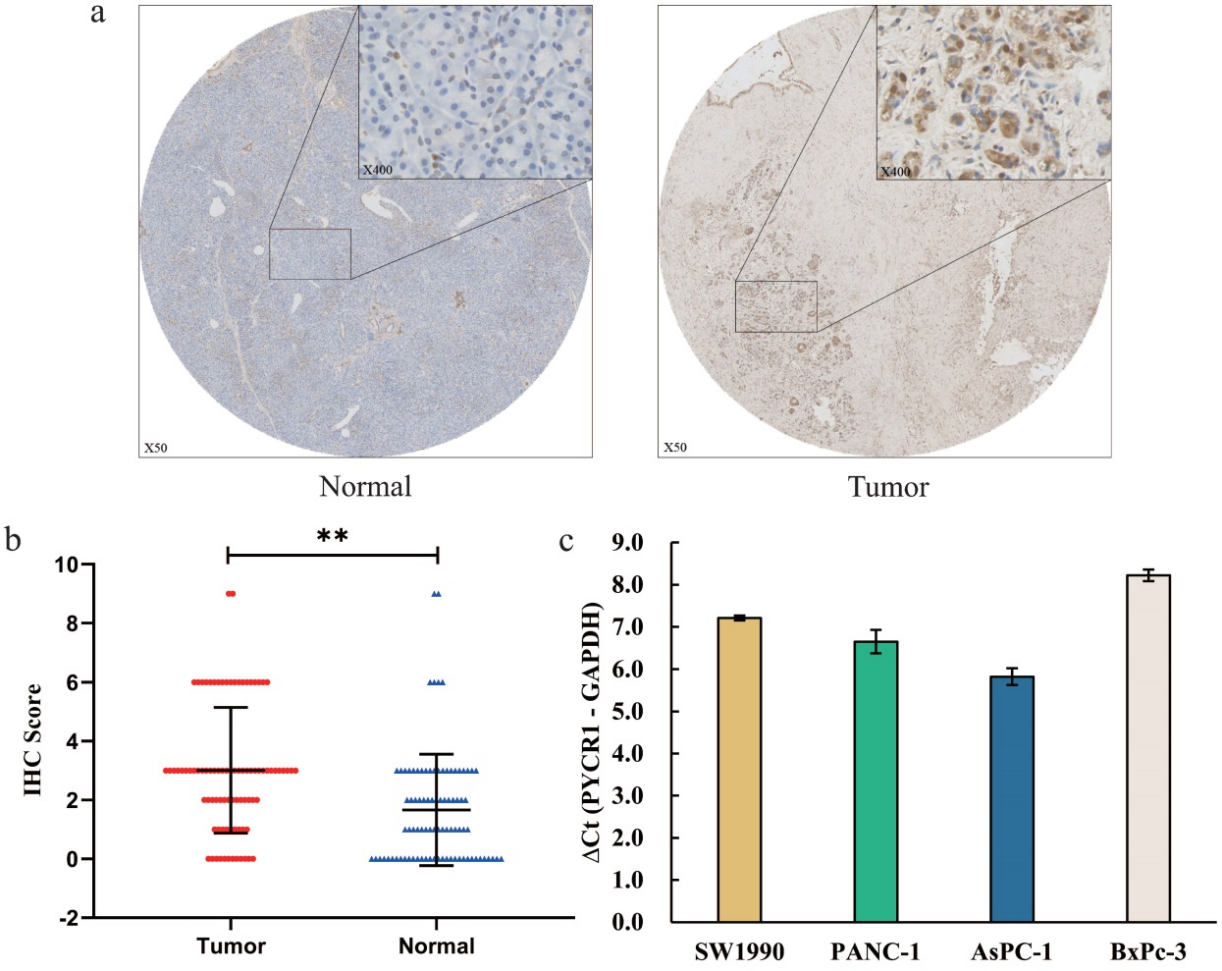
** The expression of PYCR1 in pancreatic cancer tissues and cells. (a. b)** The relative expression and IHC scores of PYCR1 in 89 PDAC tissues. The PYCR1 expression was significantly higher in tumor tissues than normal pancreatic tissues. **(c)** The mRNA expression of PYCR1 in four pancreatic cell lines. The RT-qPCT showed that the mRNA expression of PYCR1 was high in all cell lines. ΔCt = Ct value of PYCR1 - Ct value of GAPDH. Cells with larger ΔCt have lower abundance of target gene. * P<0.05, ** P<0.01.

To evaluate PYCR1 protein expression in PDAC patients, immunohistochemical staining was performed to measure the expression of PYCR1 in 89 paraffin-embedded PDCA tissues and paired normal adjacent tissues. Our results revealed that PYCR1 expression was higher in tumor tissues compared with that in the adjacent normal tissues (Fig. 1a). Meanwhile, IHC scores of PYCR1 in tumor tissues were significantly greater than in the paired normal tissues (Tumor vs Normal = 3.0 ± 2.1 vs 1.7 ± 1.9, P <0.001, Fig. 1b).

Furthermore, the mRNA expression of PYCR1 was detected by RT-qPCR in four pancreatic cell lines (SW1990, PANC-1, AsPC-1, BxPc-3). Relative abundances are shown as average ΔCt for low abundance (ΔCt ≥ 16), moderate abundance (12 < ΔCt < 16), and high abundance (ΔCt ≤ 12). The result showed that the mRNA expression abundance of PYCR1 was high in all pancreatic cell lines (SW1990 ΔCt = 7.21, PANC-1 ΔCt = 6.65, AsPC-1 ΔCt = 5.82, BxPc-3 ΔCt = 8.22; Fig. 1c). For further study, we selected PANC-1 and AsPC-1 cell lines with higher expression of PYCR1 based on the results.

### The correlation between elevated PYCR1 expression and poor prognosis of PDAC patients

To assess the correlation between the PYCR1 expression level and clinicopathological features in PDAC patients, we divided the 89 patients into high expression group (n=54) and low expression group (n=35) according to the IHC scores of PYCR1 expression. The representative IHC staining of PYCR1 in PDAC tissues and normal pancreatic tissues is shown in Figure 1a. And we analyzed the relationship between PYCR1 expression and the clinicopathological characteristics (Table 1). There was no significant association between PYCR1 expression and the clinicopathologic features. Nevertheless, patients with high expression of PYCR1 showed an increasing but non-significant trend in regional lymph node metastases and poorer differentiation. Furthermore, univariate and multivariate Cox survival analyses were performed on PYCR1 expression and the clinicopathological features (Table 2). The univariate Cox regression revealed that poorer differentiation, regional lymph node metastases, and higher expression of PYCR1 were significant risk factors for overall survival (OS) in PDAC patients (P<0.05). And PDAC patients with high PYCR1 expression had significantly shorter median OS than those with low PYCR1 expression (27.9 months vs 45.7 months, P=0.013). The multivariate Cox regression indicated that poorer differentiation (HR: 2.601, 95%CI: 1.304-5.187, P=0.007) and regional lymph node metastases (HR: 3.133, 95% CI: 1.622-6.055, P<0.001) were independent prognostic factors of OS. Moreover, high PYCR1 expression was an independent prognostic factor for OS in PDAC patients as well (HR: 2.086, 95% CI: 1.151-3.779, P=0.015).

**Figure 2:**
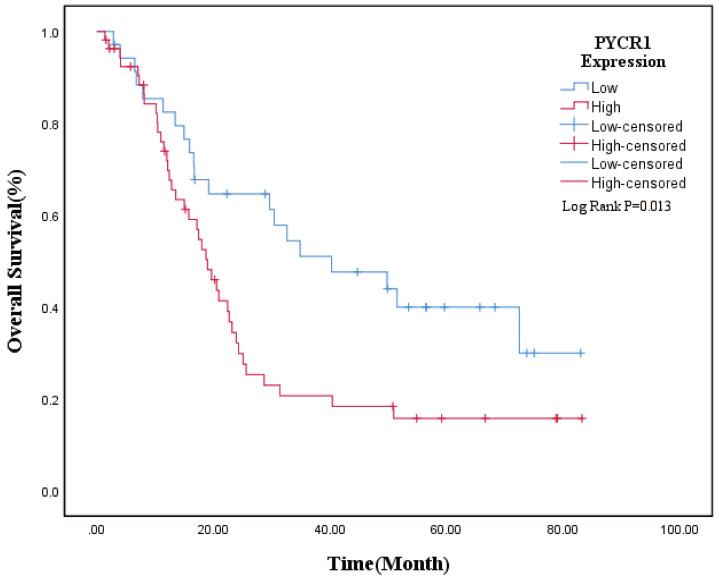
** The overall survival curve in PDAC patients.** PDAC patients with low expression of PYCR1 (n=35) had longer OS than those with high expression of PYCR1 (n=54) (P<0.05).

And the Kaplan-Meier survival curve was plotted to evaluate the prognostic value of PYCR1. The results revealed that high expression of PYCR1 had an important effect on the OS of PDAC patients. Patients with high PYCR1 levels had significantly longer OS than the low counterparts (Low expression vs High expression = 45.74±5.47 months vs 27.90±3.82 months, P=0.013; Fig. 2). Collectively, the results suggest that PYCR1 is upregulated in PDAC cells and tissues and predicts a poor prognosis in PDAC patients.

### PYCR1 expression is efficiently suppressed in PDAC cells

**Figure 3:**
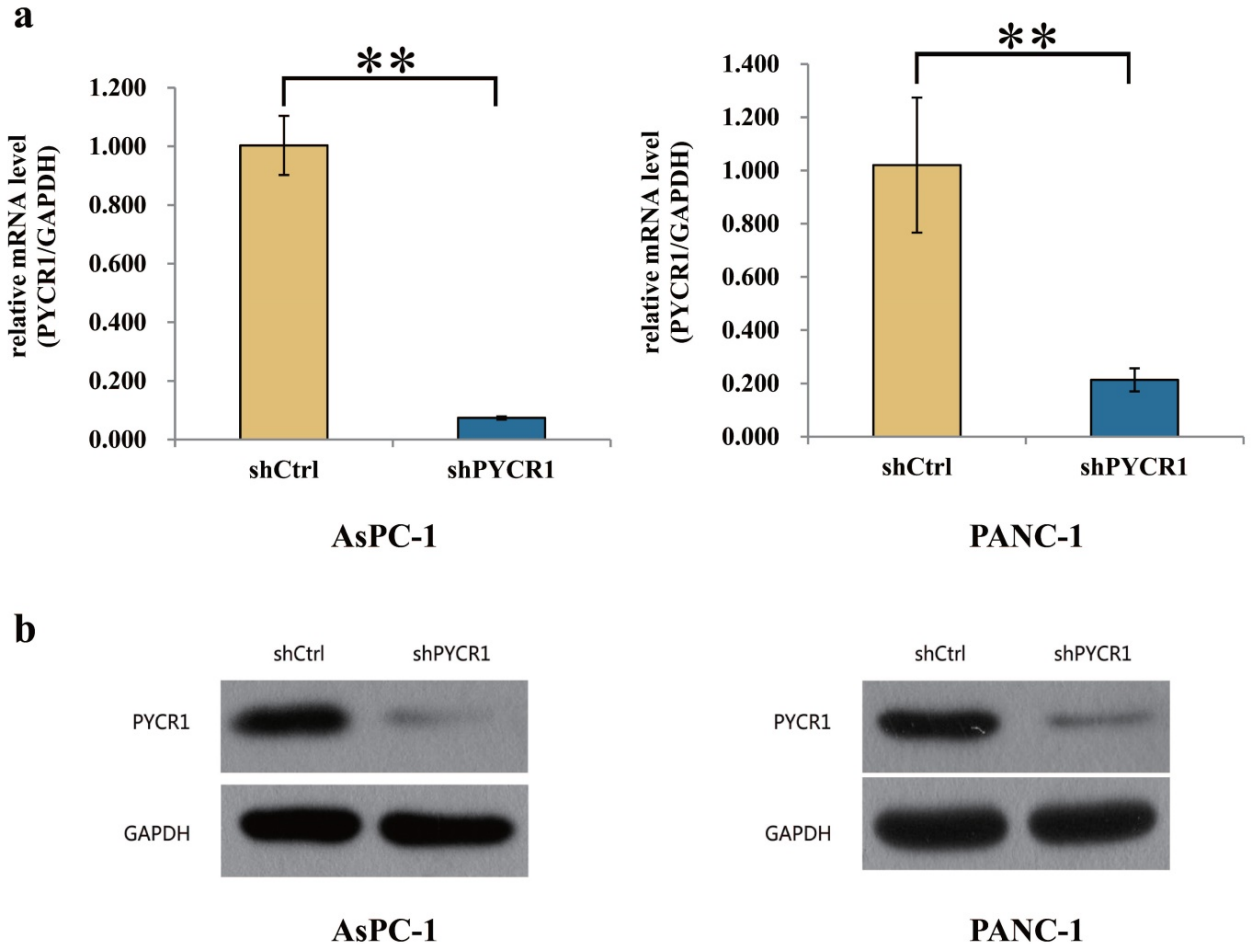
Interference efficiency of PYCR1 in AsPC-1 and PANC-1 cell lines. **(a)** Compared with shCtrl group, the mRNA expression of PYCR1 was significantly suppressed in shPYCR1 group after infected by the lentivirus. **(b)** The result of Western Blot showed that the protein expression of PYCR1 was significantly inhibited in shPYCR1 group. * P<0.05, ** P<0.01.

Before proceeding to explore the effect of PYCR1 *in vitro*, we treated the PANC-1 and AsPC-1 cells with shCtrl or shPYCR1 lentivirus. Then the level of mRNA and protein was assayed for PYCR1 using RT-qPCR and Western blot 3 days after the lentivirus infection. Compared with those in the shCtrl group, the mRNA expression of PYCR1 in the shPYCR1 group was significantly inhibited (AsPC-1, 92.6% knockdown, shCtrl vs shPYCR1 = 1.003±0.101 vs 0.074±0.005, P=0.004; PANC-1, 78.6% knockdown, shCtrl vs shPYCR1 = 1.020±0.254 vs 0.214±0.043, P=0.006; Fig. 3a). Meanwhile, the result of Western blot also indicated that the protein expression level in the shPYCR1 group was significantly suppressed (Fig. 3b).

### PYCR1 enhances the proliferation and inhibits the apoptosis of PDAC cell lines *in vitro*

**Figure 4:**
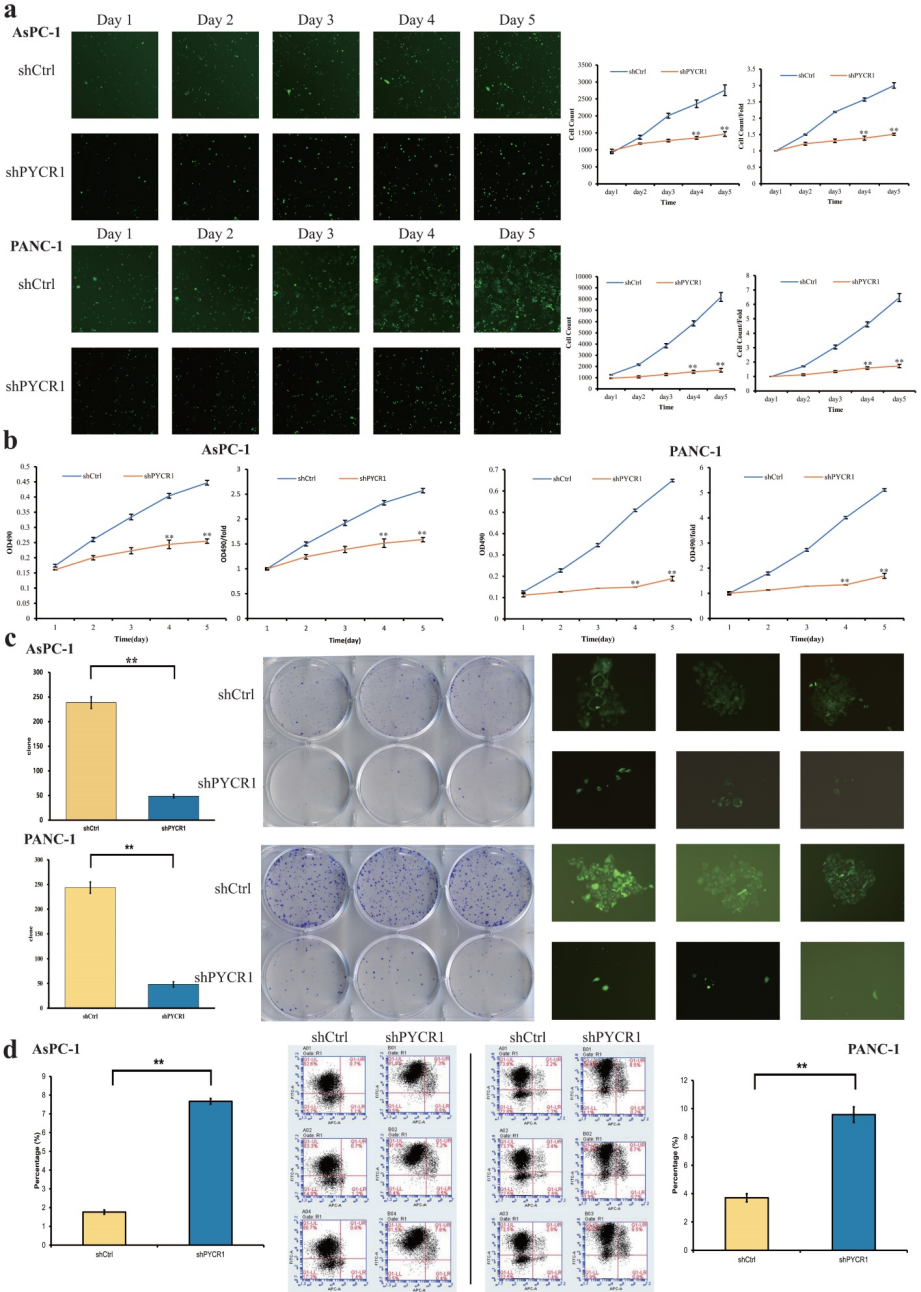
** Effect of PYCR1 interference *in vitro*. (a)** Celigo assay. The proliferation rate of AsPC-1 and PANC-1 cells was significantly decreased after PYCR1 interference. **(b)** MTT assay. The proliferation rate of AsPC-1 and PANC-1 cells was significantly inhibited in shPYCR1 group. **(c)** Colony formation assay. Interference of PYCR1 reduced colony formation significantly in AsPC-1 and PANC-1 cells. **(d)** Apoptosis assay. Percentage of apoptotic cells in shPYCR1 group was significantly increased after PYCR1 interference. Each experiment was triplicated. * P<0.05, ** P<0.01.

After infection, the proliferation of PDAC cells was analyzed by Celigo and MTT assays. The results were plotted into line graphs and the results of first day were used as controls. The results showed that the inhibition of cell growth and proliferation was significant in the shPYCR1 group, respectively. (Celigo/Cell count/Day5: AsPC-1, shCtrl vs shPYCR1 = 2757±157 vs 1467±68, P<0.001; PANC-1, shCtrl vs shPYCR1 = 8183±397 vs 1661±162, P<0.001; Fig. 4a) (MTT/OD490/Day5: AsPC-1, shCtrl vs shPYCR1 = 0.447±0.008 vs 0.255±0.007, P<0.001; PANC-1, shCtrl vs shPYCR1 = 0.649±0.006 vs 0.189±0.011, P<0.001; Fig. 4b). Simultaneously, the colony formation assay was performed and the number of colonies in both groups is shown in bar graphs. Results demonstrated that the capacity of the shPYCR1 group to form colonies was decreased significantly compared with the shCtrl group (AsPC-1, shCtrl vs shPYCR1 = 239±12 vs 49±4, P<0.001; PANC-1, shCtrl vs shPYCR1 = 244±12 vs 48±6, P<0.001; Fig. 4c). Further cell apoptosis detection by flow cytometry revealed that the percentage of apoptosis was increased significantly in the shPYCR1 group (AsPC-1, shCtrl vs shPYCR1 = 1.76±0.11 vs 7.67±0.15, P<0.001; PANC-1, shCtrl vs shPYCR1 = 3.71±0.29 vs 9.58±0.55, P<0.001; Fig. 4d). Taken together, the data suggest that PYCR1 promotes PDAC cell proliferation and suppresses cell apoptosis.

### PYCR1 promotes tumor growth *in vivo*

**Figure 5:**
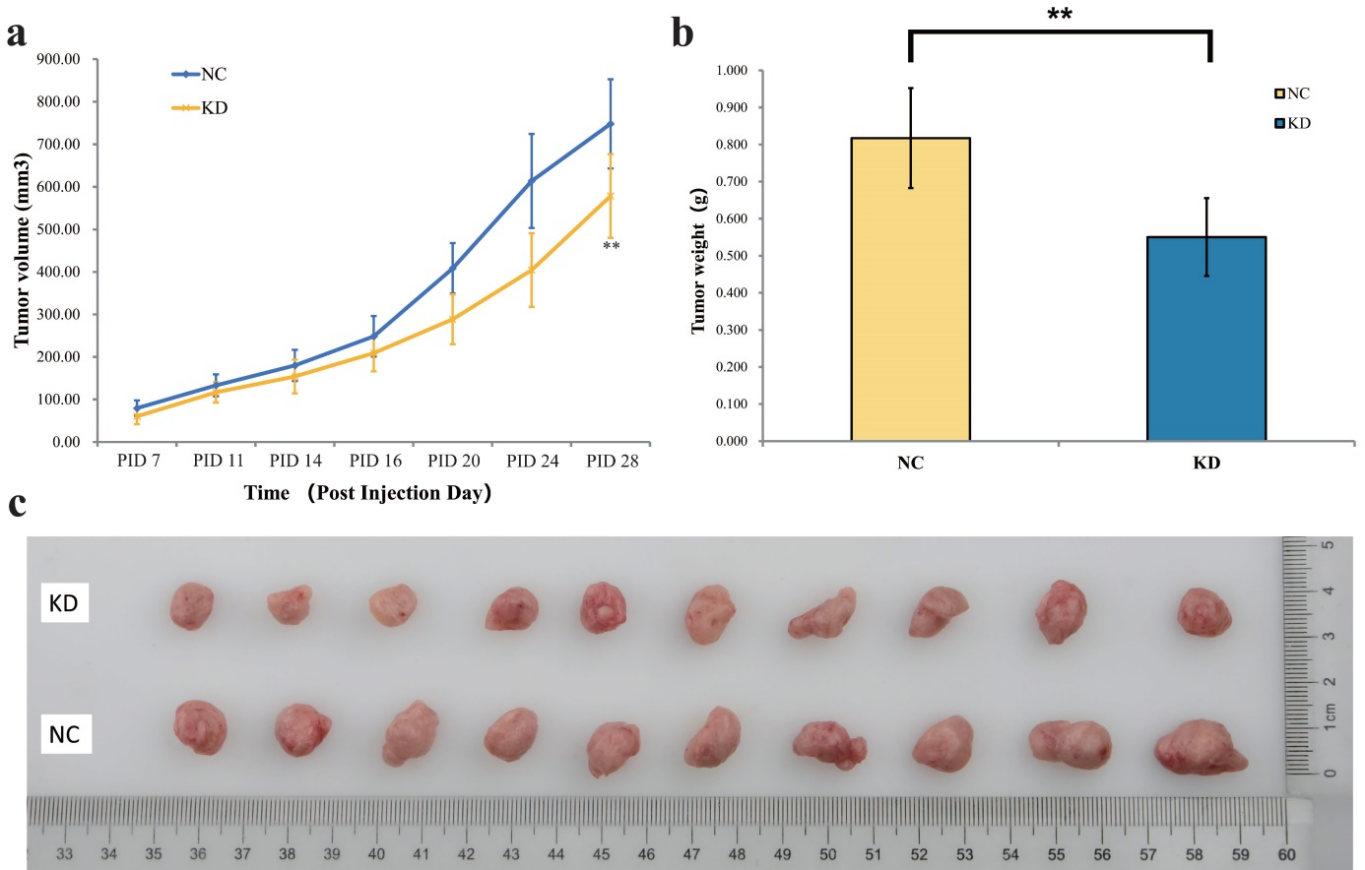
** Effect of PYCR1 interference *in vivo*. (a)** Change in tumor volume. The average tumor volume in the NC group was significantly larger. Tumor volume = π/6 × Length × Width × Width. **(b)** Change in tumor weight. The tumor in the NC group was heavier than that in the KD group. **(c)** Images of the dissected tumors from mice. * P<0.05, ** P<0.01.

In the final part of the study, the AsPC-1 cells were injected subcutaneously into nude mice to explore the effect of PYCR1 *in vivo*. The AsPC-1 cells were treated with shPYCR1 or shCtrl lentivirus. The average tumor volume in the NC group was significantly larger than that in the KD group (NC vs KD = 748.07±104.83 mm^3^ vs 578.25±98.43 mm^3^, P=0.002; Fig. 5a). Moreover, the average tumor weight in the NC group was significantly increased compared with the KD group (NC vs KD = 0.817±0.135 g vs 0.551±0.105 g, P<0.001; Fig. 5b). After the Mice were euthanized, the tumors were weighed and photographed (Fig. 5c).

## Discussion

Glutamine is an important energy source for cancer cells and the metabolic disorders of glutamine are closely related to tumorigenesis and cancer progression. Glutamine converses into glutamate in the first step of glutamine metabolism. In particular, glutamate, a key role both in glutamine metabolism and tumorigenesis, is one of the most significant sources for the synthesis of proline 24,25. Therefore, the role of the proline synthesis in tumorigenesis and cancer progression has gained increasing attention in recent years. PYCR, a mitochondrial matrix enzyme widely expressed in human tissues, is essential for the synthesis of proline. The PYCR family comprises the isozymes PYCR1, PYCR2, and PYCRL 25,26. Many studies have demonstrated the overexpression of PYCR1 in several kinds of tumor tissues and the importance of PYCR1 in tumorigenesis and cancer progression 20-23,27. However, the expression and function of PYCR1 in pancreatic cancer remain unknown. The initial objective of our study is to identify the effect of PYCR1 on PDAC.

The study found that the expression of PYCR1 in 89 tumor tissues was significantly higher than that in the paired adjacent normal tissues. And the further analysis confirmed that the overexpression of PYCR1 may be associated with pancreatic cancer occurrence and progression. The results showed that the median OS of the high PYCR1 expression group was only 27.9 months, while the median OS of the low PYCR1 expression group was 45.7 months. Multivariate Cox regression analysis in 89 patients revealed that the high PYCR1 expression level was an independent prognostic factor predicting worse OS for PDAC patients. Based on the above study, we hypothesized that there may be a close relationship between PDAC tumorigenesis, progression, and PYCR1 expression. To confirm our discovery, we further investigated the role of PYCR1 both *in vitro* and *in vivo*. Celigo assay, MTT assay, and colony formation assay showed that the pancreatic cells infected by lentivirus grew slowly. And the results of the flow cytometry showed the percentage of apoptotic cells was decreased in the infected cells. Additionally, PYCR1 interference significantly suppressed tumor growth in nude mice. Taken together, all data demonstrated that PYCR1 plays an essential role in tumorigenesis and progression of PDAC cells.

These results corroborate the findings of a great deal of the previous work on the effect of PYCR1 on tumors. A previous study, which collected about 2500 cases from 13 independent microarray datasets, found that PYCR1 was overexpressed in breast cancer. The PYCR1 interference significantly suppressed the breast cancer cell growth and invasion by regulating AKT/ERK signaling pathway. And it enhanced the cytotoxicity of doxorubicin in breast cancer cell lines 28,29. Another study confirmed that PYCR1 was negatively regulated by miR-488 and promoted the occurrence and development of non-small cell lung cancer by activating the p38mapk pathway. The up-regulation of PYCR1 significantly increased the expression of p38 and promoted the nuclear accumulation of p38. Further, the expression of PYCR1 was negatively regulated by miR-488, the upregulation of which inhabited the cell proliferation, increased apoptosis and decreased p38 expression and nuclear accumulation. Besides, research by Gao et al, found that the interference of PYCR1 increased the expression of Bcl-2 and c-Myc, and the phosphorylation level of JAK2 and STAT3 in lung adenocarcinoma, which further affected the JAK/STAT signaling pathway 27,30,31. And a study of papillary renal cell carcinoma demonstrated that phosphorylates Akt (p-Akt) and phosphorylates mTOR (p-mTOR) were inhibited by the suppression of PYCR1. The result indicated that PYCR1 may regulate the progression of papillary renal cell carcinoma through the Akt/mTOR pathway 20,22. In prostate cancer, previous studies revealed the possible mechanism of PYCR1 in tumorigenesis. After PYCR1 knockdown, the expression of cell cycle-regulating proteins was down-regulated, while the expression of apoptosis-related proteins increased 32. These are particularly encouraging findings that PYCR1 plays a vital role in tumor initiation and progression 33-35.

## Conclusion

In summary, according to our preliminary results of the relationship between PYCR1 and PDAC, PYCR1 interference leads to inhibition of proliferation and promotion of apoptosis. It will provide a potential biomarker of diagnosis, prognosis, and therapy for pancreatic cancer. However, more detailed studies are needed to confirm the mechanism of PYCR1 on pancreatic cancer. This remains a goal of our further research.

## Figures and Tables

**Table 1:** Relationship between clinicopathological data and PYCR1 expression of in PDAC patients

Variable	PYCR1 Expression		P-value
	Low (n=35)	High (n=54)	
**Age at onset, n (%)**			0.332
<60 years	11(31.4)	12(22.2)	
≥60years	24(68.6)	42(77.8)	
**Sex, n (%)**			0.151
Male	18(51.4)	36(66.7)	
Female	17(48.6)	18(33.3)	
**Tumor size, n (%)**			0.242
≤3 cm	15(42.9)	30(55.6)	
>3 cm	20(57.1)	24(44.4)	
**Location, n (%)**			0.772
Head	19(54.3)	31(57.4)	
Distal	16(45.7)	23(42.6)	
**TNM stage, n (%)**			0.344
I	1(2.9)	3(5.6)	
II	25(71.4)	41(75.9)	
III	9(25.7)	10(18.5)	
**Tumor differentiation, n (%)**			0.261
Well, moderate	13(37.1)	14(25.9)	
Poor	22(62.9)	40(74.1)	
Neural invasion, n (%)	35(100.0)	48(88.9)	0.108
Vascular invasion, n (%)	2(5.7)	1(1.9)	0.700
Regional lymph node metastases, n (%)	12(34.3)	23(42.6)	0.433
**CA19-9, n (%)**			0.915
≤37 U/ml	10(28.6)	16(29.6)	
>37 U/ml	25(71.4)	38(70.4)	

**Table 2:** Overall survival analysis of PDAC patients on univariate and multivariate analysis

Variable	Univariate analysis			Multivariate analysis	
	No. of cases	Mean survival (months)	P‑value	HR (95% CI)	P‑value
**Age at onset**			0.617		
<60 years	23	32.40			
≥60 years	66	36.32			
**Sex**			0.727		
Male	54	34.20			
Female	35	37.27			
**Tumor size**			0.969		
≤3 cm	45	36.64			
>3 cm	44	32.83			
**Location**			0.429		
Head	50	33.47			
Distal	39	36.83			
**TNM stage**			0.284		
I	4	59.76			
II	66	31.39			
III	19	41.24			
**Tumor differentiation**			0.002	2.601(1.304-5.187)	0.007
Well, moderate	27	50.15			
Poor	62	27.11			
**Neural invasion**			0.886		
Absent	6	31.47			
Present	83	35.58			
**Vascular invasion**			0.954		
Absent	86	35.32			
Present	3	32.29			
**Regional lymph node metastases**			<0.001	3.133(1.622-6.055)	<0.001
Absent	54	43.77			
Present	35	20.63			
**CA19-9**			0.701		
≤37 U/ml	26	35.50			
>37 U/ml	63	34.80			
**PYCR1 expression**			0.013	2.086(1.151-3.779)	0.015
Low expression	35	45.74			
High expression	54	27.89			
